# Incidence, Patterns, and Predictive Factors of Biochemical Failure Following Definitive Radiotherapy for Localized Prostate Cancer: A Retrospective Military Cohort Study

**DOI:** 10.7759/cureus.115697

**Published:** 2026-09-03

**Authors:** Wajdi Alnajada, Nizar M Ghnmein, Rawan Ayyad, Abdullah Abukaff, Maher Bani Essa, Laith Alhabahbeh, Anfal N Daheerat, Ashraf Alzyoud, Zaid Alhiary

**Affiliations:** 1 Department of Radiation Oncology, Military Cancer Center, Royal Medical Services, Amman, JOR; 2 Department of Surgical Oncology, Military Cancer Center, Royal Medical Services, Amman, JOR; 3 Department of Hematology, Military Cancer Center, Royal Medical Services, Amman, JOR

**Keywords:** biochemical recurrence, jordan, prostate-specific antigen (psa), prostatic neoplasms, radiotherapy (rt)

## Abstract

Background: Definitive radiotherapy represents a cornerstone treatment for localized prostate cancer, offering curative potential comparable to surgery. However, biochemical failure remains a concern, signaling possible disease progression.

Objective: This study aimed to evaluate the frequency, timing, and predictors of biochemical failure in patients receiving definitive radiotherapy for localized prostate cancer at a military oncology center.

Methods: We conducted a retrospective cohort study of 91 patients with localized or locally advanced prostate cancer (cT1-T3, N0-1, M0) treated with definitive radiotherapy at the Military Cancer Center in Jordan between 2020 and 2025. Biochemical failure was defined using the Phoenix criteria (prostate-specific antigen (PSA) nadir + 2 ng/mL). Biochemical recurrence-free survival was estimated using Kaplan-Meier analysis, and independent predictors of biochemical failure were identified using Cox proportional hazards regression.

Results: The median age was 70 years, and the median pre-treatment PSA was 18.0 ng/mL. According to National Comprehensive Cancer Network (NCCN) risk stratification, 62/91 (68.1%) had high/very high-risk disease, 25/91 (27.5%) had intermediate-risk disease, and 4/91 (4.4%) had low-risk disease. Over a median follow-up of 36 months, 22 patients (24.2%) experienced biochemical failure, with a median time to failure of 24 months. The two-year and five-year biochemical recurrence-free survival rates were 84.6% and 70.3%, respectively. Multivariable analysis identified pre-treatment PSA greater than 20 ng/mL (hazard ratio (HR): 2.8; 95% confidence interval (CI): 1.4-5.6; p = 0.004) and PSA nadir greater than 0.1 ng/mL (HR: 3.2; 95% CI: 1.6-6.4; p = 0.001) as the strongest independent predictors of biochemical failure. Patients with PSA nadir > 0.1 ng/mL had a 3.2-fold higher hazard of biochemical failure compared to those with PSA nadir ≤ 0.1 ng/mL.

Conclusions: Definitive radiotherapy provides favorable biochemical control in this military cohort, with outcomes comparable to international benchmarks. Pre-treatment PSA and post-treatment PSA nadir independently predict biochemical failure, supporting their role in risk stratification and individualized follow-up planning. However, these findings require prospective validation in larger, multicenter cohorts before individualized surveillance strategies can be widely implemented.

## Introduction

Prostate cancer ranks as the fourth most commonly diagnosed malignancy worldwide, following lung, female breast, and colorectal cancers. However, it remains the second most frequently diagnosed cancer in men globally, after lung cancer [1]. In Jordan, prostate cancer incidence has shown a consistent upward trend over recent decades, driven by population aging, improved diagnostic techniques, and more comprehensive cancer registration [2].

Definitive radiotherapy has become a primary treatment option for localized prostate cancer, providing cure rates comparable to radical prostatectomy while avoiding surgical risks [3,4]. Modern radiotherapy approaches, including intensity-modulated radiation therapy (IMRT), volumetric modulated arc therapy (VMAT), and stereotactic body radiation therapy (SBRT), enable precise tumor targeting while minimizing exposure to surrounding healthy tissues [5].

Despite excellent initial responses, some patients experience recurrence, which often presents as a rising prostate-specific antigen (PSA) without detectable clinical or radiographic disease-a condition known as biochemical failure [6]. The Phoenix definition, established at the 2005 Radiation Therapy Oncology Group-American Society for Therapeutic Radiology and Oncology (RTOG-ASTRO) consensus conference, defines biochemical failure as an increase of 2 ng/mL or more above the post-treatment PSA nadir [6]. This definition has been universally adopted for reporting outcomes after definitive radiotherapy [7].

Multiple factors contribute to biochemical failure risk, including tumor biology, treatment characteristics, and patient-specific factors. Well-established pre-treatment prognostic indicators include baseline PSA level, Gleason score, clinical T-stage, and perineural invasion [8,9]. Treatment-related factors such as radiation dose, fractionation schedule, and the use of androgen deprivation therapy (ADT) substantially affect outcomes [10,11]. Post-treatment parameters, particularly PSA nadir and PSA kinetics, offer valuable insights into treatment efficacy and residual disease burden [12].

The military healthcare system provides unique opportunities for longitudinal prostate cancer research due to its integrated care model, standardized treatment protocols, comprehensive electronic medical records, and minimal patient loss to follow-up. Previous reports from military treatment centers have demonstrated outcomes comparable to or better than those from academic tertiary institutions [13,14].

This study addresses a significant knowledge gap by providing a detailed analysis of biochemical failure patterns in a Middle Eastern military population. Our objectives were to (1) determine the incidence and timing of biochemical failure after definitive radiotherapy, (2) identify baseline and treatment-related predictors of biochemical failure, (3) estimate biochemical recurrence-free survival (bRFS) stratified by clinical risk categories, and (4) establish local benchmarks to inform clinical practice.

## Materials and methods

Ethics statement

This study received ethical approval from the Institutional Review Board (IRB) of the Jordanian Royal Medical Services (Registration Number: 39_1/2026) on January 12, 2026. On March 9, 2026, the Jordanian Royal Medical Services Educational and Training Directorate provided final authorization for data dissemination and publication. The study was conducted in accordance with the ethical principles outlined in the Declaration of Helsinki. The IRB granted a waiver for individual informed consent due to the retrospective, anonymized nature of the research.

Study design and setting

We conducted a retrospective cohort study at the Department of Radiation Oncology, Military Cancer Center, Jordanian Royal Medical Services, in Amman, Jordan, covering the period from January 2020 to June 2025. The study follows the Strengthening the Reporting of Observational Studies in Epidemiology (STROBE) guidelines for observational cohort studies [15].

Study population

The study population included all patients with localized or locally advanced prostate cancer who were treated with definitive radiotherapy at the Military Cancer Center during the study period. Patients were eligible for inclusion if they had histologically confirmed prostate adenocarcinoma, presented with localized (cT1-T2, N0, M0) or locally advanced (cT3-T4, N0-1, M0) disease according to the American Joint Committee on Cancer (AJCC) 8th edition staging system [16], completed definitive external beam radiotherapy (EBRT) with curative intent, were aged 18 years or older, had at least three post-treatment PSA measurements available, and had a minimum follow-up of six months after radiotherapy completion.

Patients were excluded if they had metastatic disease (M1) at presentation, had previously received pelvic radiotherapy or undergone radical prostatectomy, had incomplete radiation treatment (less than 90% of the prescribed dose), had a synchronous second malignancy, had insufficient follow-up data, or had been treated with primary brachytherapy alone. Following the application of these criteria, the final analytical cohort comprised 91 patients.

Data collection

We extracted comprehensive data from electronic medical records using a standardized data collection form. Variables collected included age, pre-treatment PSA level, biopsy Gleason score (converted to grade groups according to the 2014 International Society of Urological Pathology (ISUP) consensus) [17], clinical T-stage, nodal status, metastatic status, National Comprehensive Cancer Network (NCCN) risk group classification (high/very high-risk combined) [18], comorbidity assessment, radiotherapy technique and dose, fractionation schedule, treatment volume, ADT details, and follow-up PSA measurements.

Radiotherapy

All patients received definitive EBRT using IMRT or VMAT with conventional fractionation. SBRT and hypofractionated regimens were not used in this cohort. The total dose ranged from 6,000 to 7,920 cGy (median: 6,800 cGy), delivered in 25-40 fractions (median: 27 fractions), with a dose per fraction of 200-240 cGy. The target volume included the prostate with or without the seminal vesicles, depending on risk group. A clinical target volume to planning target volume (CTV-PTV) margin of 5-8 mm was applied. Daily image guidance using cone-beam computed tomography (CBCT) was performed for all patients. Table 1 summarizes the stage-wise radiotherapy doses, fractionation schedules, and ADT regimens.

**Table 1:** Stage-Wise Radiotherapy Dose, Fractionation, and ADT Regimens ADT: androgen deprivation therapy; IMRT: intensity-modulated radiation therapy; VMAT: volumetric modulated arc therapy; cGy: centigray

Risk group	Total dose (cGy)	Fractions	Dose per fraction (cGy)	Technique	ADT duration
Low risk	7,000-7,200	35-36	200	IMRT/VMAT	None or 4 months
Intermediate risk	7,200-7,600	36-38	200	IMRT/VMAT	4-6 months
High/very high risk	7,600-7,920	38-40	200-240	IMRT/VMAT	6-24 months

Androgen Deprivation Therapy

ADT was given as depot injections every three months using luteinizing hormone-releasing hormone (LHRH) agonists (leuprolide acetate 22.5 mg or goserelin acetate 10.8 mg). ADT was initiated 2-3 months prior to radiotherapy (neoadjuvant) and continued during radiotherapy. For high/very high-risk patients, ADT was continued after radiotherapy completion (adjuvant). The median duration of ADT was six months (range: 4-24 months), with intermediate-risk patients receiving 4-6 months and high/very high-risk patients receiving 6-24 months.

Outcome assessment

The primary outcome was biochemical failure, defined according to the Phoenix consensus criteria as a PSA rise of 2 ng/mL or more above the post-treatment nadir [6]. The date of the first PSA value that met this definition was recorded as the date of biochemical failure. PSA nadir was defined as the lowest post-treatment PSA value achieved after radiotherapy completion, and the time to nadir was documented. The nadir threshold of 0.1 ng/mL was used for risk stratification based on literature evidence [19,20]. Secondary outcomes included time to biochemical failure, PSA nadir value, bRFS at two and five years, and salvage treatment utilization.

Statistical analysis

All statistical analyses were performed using R software version 4.3.2 (R Foundation for Statistical Computing, Vienna, Austria). A two-tailed p-value less than 0.05 was considered statistically significant.

Continuous variables are displayed as medians accompanied by interquartile ranges (IQRs), whereas categorical variables are shown as frequencies and percentages. bRFS was calculated from the date of radiotherapy completion to the date of biochemical failure or last follow-up, estimated using the Kaplan-Meier method, with comparisons between groups performed using the log-rank test. Univariate Cox proportional hazards regression models were fitted to examine associations between potential predictors and biochemical failure. Variables with p < 0.10 in univariate analysis, along with clinically significant variables, were considered for inclusion in multivariate models. Backward stepwise selection with Akaike Information Criterion (AIC) optimization was employed to identify independent predictors. Results are presented as hazard ratios (HRs) with 95% confidence intervals (CIs).

## Results

Patient characteristics

Table 2 presents the baseline demographic and clinical characteristics of the 91 patients. The median age was 70 years (range 51-90). The median pre-treatment PSA was 18.0 ng/mL (IQR: 11.1-34.8). According to NCCN risk stratification, 62/91 (68.1%) had high/very high-risk disease, 25/91 (27.5%) had intermediate-risk disease, and 4/91 (4.4%) had low-risk disease. Gleason score 7 (grade groups 2-3) was observed in 44/91 (48.4%), and Gleason score 8-10 (grade groups 4-5) in 32/91 (35.2%). Locally advanced disease (T3-T4) was present in 33/91 (36.3%) of patients.

**Table 2:** Baseline Patient Characteristics (N = 91) PSA: prostate-specific antigen; ADT: androgen deprivation therapy; IQR: interquartile range; NCCN: National Comprehensive Cancer Network; IMRT: intensity-modulated radiation therapy; VMAT: volumetric modulated arc therapy; IGRT: image-guided radiation therapy; CBCT: cone-beam computed tomography

Characteristic	Value
Age (years), median (IQR)	70 (64-75)
Pre-treatment PSA (ng/mL), median (IQR)	18.0 (11.1-34.8)
PSA > 20 ng/mL, n (%)	39 (42.9%)
Gleason score ≥ 8, n (%)	32 (35.2%)
T3-T4 disease (locally advanced), n (%)	33 (36.3%)
N1 disease, n (%)	19 (20.9%)
NCCN risk group, n (%)	
Low risk	4 (4.4%)
Intermediate risk	25 (27.5%)
High/very high risk	62 (68.1%)
Radiotherapy	
Technique	IMRT/VMAT
Total dose (cGy), median (IQR)	6,800 (6,000-7,000)
Dose range (cGy)	6,000-7,920
Fractions, median (range)	27 (25-40)
Dose per fraction (cGy)	200-240
IGRT	Daily CBCT
ADT use, n (%)	89 (97.8%)
ADT type, n (%)	
Leuprolide acetate	62 (69.7%)
Goserelin acetate	27 (30.3%)
ADT duration (months), median (range)	6 (4-24)

ADT was administered to 89/91 (97.8%) of patients. The primary ADT regimen used was LHRH agonists. Leuprolide acetate was administered to 62/89 (69.7%) and goserelin acetate to 27/89 (30.3%). The median duration of ADT was six months (range: 4-24 months).

The median radiotherapy dose was 6,800 cGy (range: 6,000-7,920 cGy), delivered in 25-40 fractions (median: 27 fractions), with a dose per fraction of 200-240 cGy. All patients received conventional fractionation using IMRT/VMAT with daily CBCT image guidance.

Biochemical failure incidence and timing

Over a median follow-up of 36 months, 22/91 (24.2%) patients experienced biochemical failure. The median time to biochemical failure was 24 months (IQR: 18-36 months). The majority of biochemical failure events (18/22 (81.8%)) occurred within the first 36 months following radiotherapy completion.

Biochemical recurrence-free survival

The overall bRFS for the entire cohort was 84.6% (95% CI: 77.3%-92.5%) at two years and 70.3% (95% CI: 59.8%-82.6%) at five years (Figure 1). Stratified by risk group, two-year bRFS was 100% for low-risk, 92.0% for intermediate-risk, and 80.6% for high/very high-risk patients. Five-year bRFS was 100% for low-risk, 84.0% for intermediate-risk, and 64.5% for high/very high-risk patients (Figure 2). The log-rank test confirmed significant differences in survival distributions across risk groups (p = 0.04).

**Figure 1:**
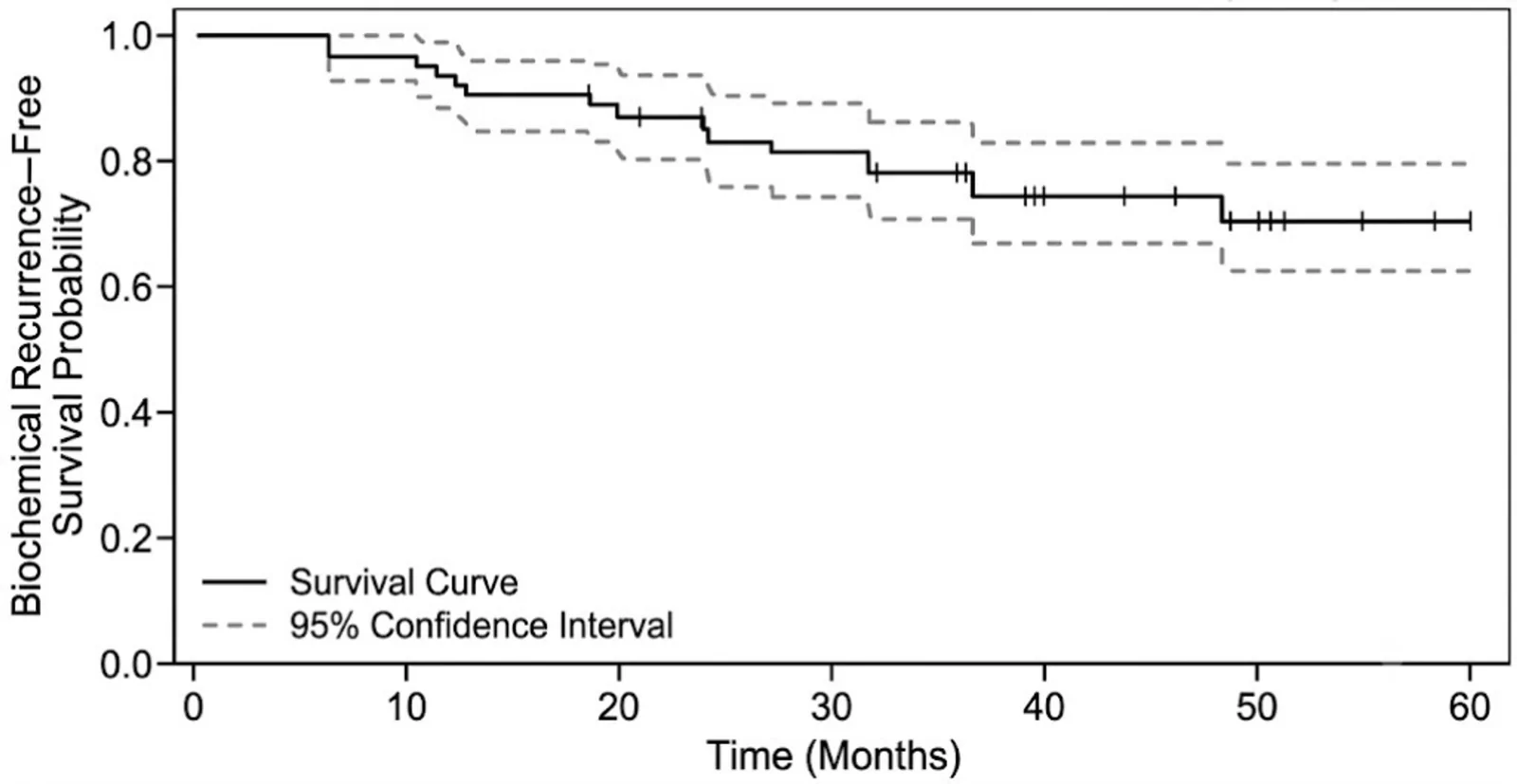
Kaplan-Meier Curve for Overall Biochemical Recurrence-Free Survival The solid line represents the survival function, with dashed lines indicating 95% CIs. Censored patients are denoted by tick marks. The 2-year biochemical recurrence-free survival was 84.6% (95% CI: 77.3%-92.5%), and the 5-year biochemical recurrence-free survival was 70.3% (95% CI: 59.8%-82.6%). CI: confidence interval

**Figure 2:**
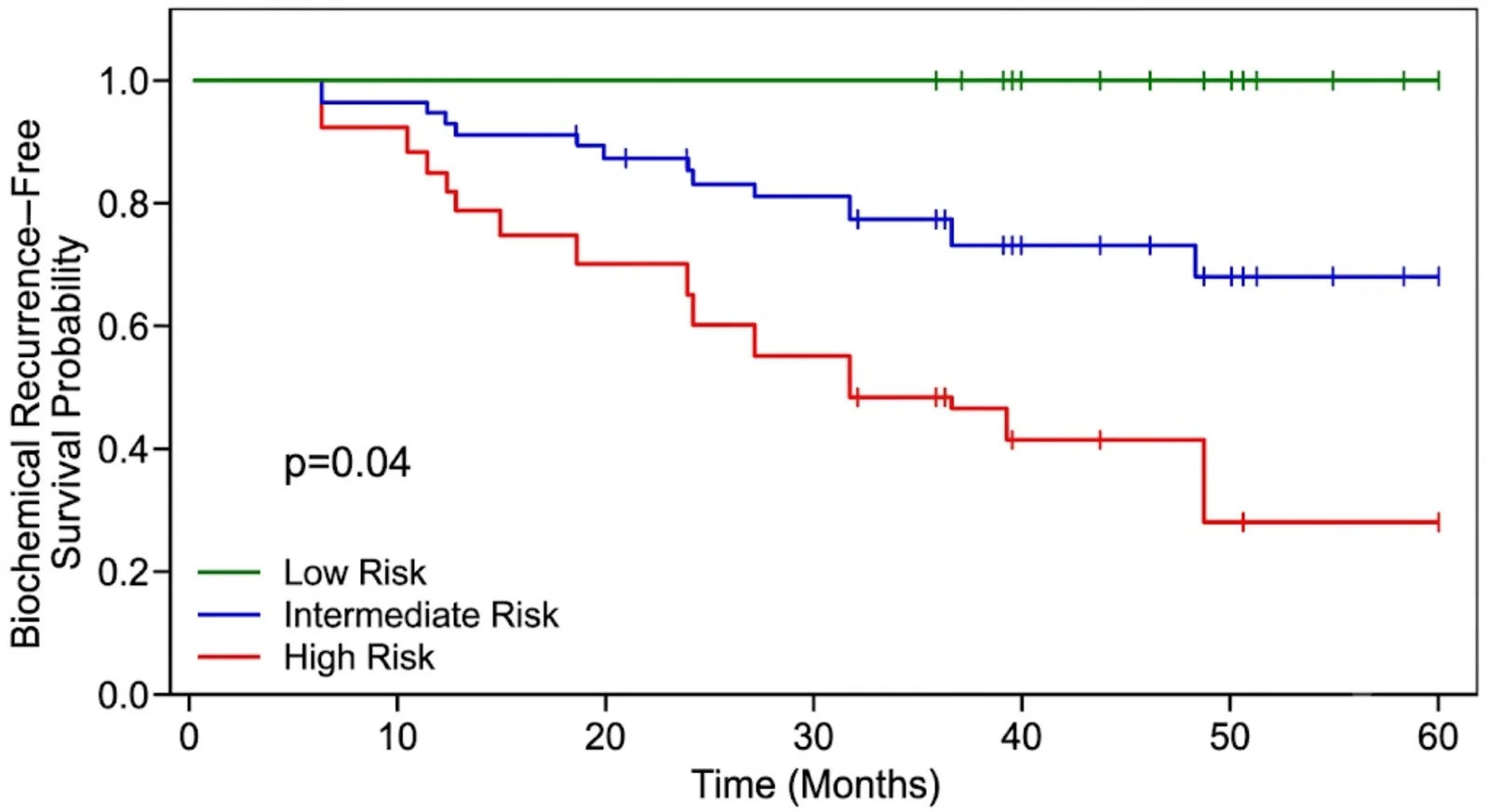
Kaplan-Meier Curves for Biochemical Recurrence-Free Survival Stratified by NCCN Risk Group The graph in this image shows the Kaplan-Meier curves for biochemical recurrence-free survival grouped by NCCN risk category. At both 2 and 5 years, low-risk patients have 100% biochemical recurrence-free survival. About 92.0% of patients with intermediate risk will not have a biochemical recurrence within 2 years, and 84.0% will not have a biochemical recurrence within 5 years. People with a high or very high risk have an 80.6% chance of not having a biochemical recurrence in 2 years and a 64.5% chance of not having one in 5 years. The log-rank test confirms that survival rates are significantly different between risk groups (p = 0.04). NCCN: National Comprehensive Cancer Network

Predictors of biochemical failure

Univariate Cox regression analysis identified pre-treatment PSA greater than 20 ng/mL (HR: 2.4; 95% CI: 1.3-4.5; p = 0.006) and PSA nadir greater than 0.1 ng/mL (HR: 3.0; 95% CI: 1.6-5.6; p < 0.001) as significant predictors of biochemical failure. Gleason score 8 or higher, N1 status, and the high/very high-risk group showed trends toward increased risk but did not reach statistical significance.

Multivariate Cox regression analysis (Table 3 and Figure 3) identified pre-treatment PSA greater than 20 ng/mL (HR: 2.8; 95% CI: 1.4-5.6; p = 0.004) and PSA nadir greater than 0.1 ng/mL (HR: 3.2; 95% CI: 1.6-6.4; p = 0.001) as independent predictors of biochemical failure. Patients with PSA nadir > 0.1 ng/mL had a 3.2-fold higher hazard of biochemical failure compared to those with PSA nadir ≤ 0.1 ng/mL.

**Table 3:** Multivariate Cox Regression Analysis for Biochemical Failure HR: hazard ratio; CI: confidence interval; PSA: prostate-specific antigen

Predictor	HR	95% CI	p-value
Pre-treatment PSA > 20 ng/mL	2.8	1.4-5.6	0.004
PSA nadir > 0.1 ng/mL	3.2	1.6-6.4	0.001

**Figure 3:**
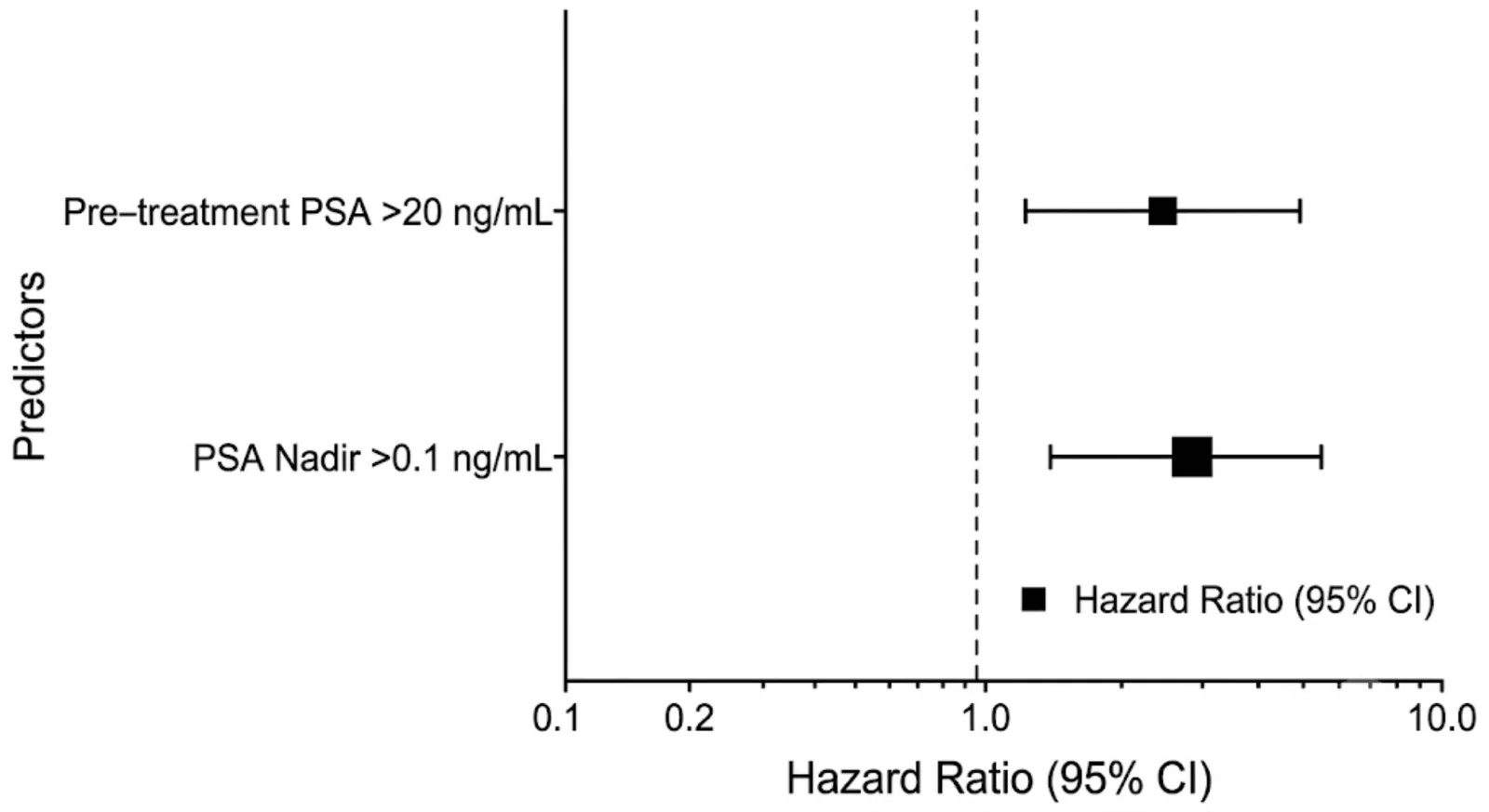
Forest Plot of Multivariate Predictors for Biochemical Failure The HRs and 95% CIs for the two independent predictors found in the multivariate Cox regression analysis are shown in this forest plot. A PSA level of more than 20 ng/mL before treatment (HR: 2.8; 95% CI: 1.4-5.6) and a PSA level of more than 0.1 ng/mL before treatment (HR: 3.2; 95% CI: 1.6-6.4) are both strong predictors, but their CIs do not go over the first value (HR = 1.0). HR: hazard ratio; CI: confidence interval; PSA: prostate-specific antigen

The median PSA nadir in our cohort was 0.08 ng/mL (IQR: 0.04-0.15 ng/mL), with a range of 0.01-0.98 ng/mL. The median time to reaching PSA nadir was 12 months (IQR: 6-18 months). In our group, 52 out of 91 (57.1%) had a PSA nadir of ≤0.1 ng/mL, and 39 out of 91 (42.9%) had a nadir of >0.1 ng/mL. Among patients with nadir ≤ 0.1 ng/mL, the biochemical failure rate was 15.4% (8/52), compared to 35.9% (14/39) in patients with nadir > 0.1 ng/mL. Table 4 presents PSA nadir distribution and biochemical failure outcomes.

**Table 4:** PSA Nadir Distribution and Biochemical Failure Outcomes PSA: prostate-specific antigen; IQR: interquartile range

Parameter	Value	
Median PSA nadir (ng/mL)	0.08 (IQR: 0.04-0.15)	
PSA nadir range (ng/mL)	0.01-0.98	
Median time to nadir (months)	12 (IQR: 6-18)	
PSA nadir category	n (%)	Biochemical failure, n (%)
≤0.1 ng/mL	52 (57.1)	8 (15.4)
>0.1 ng/mL	39 (42.9)	14 (35.9)

The model demonstrated good fit (AIC = 158.3) and met proportional hazards assumptions. Figure 4 presents a scatter plot illustrating the correlation between pre-treatment PSA and PSA nadir, with points color-coded by biochemical failure status.

**Figure 4:**
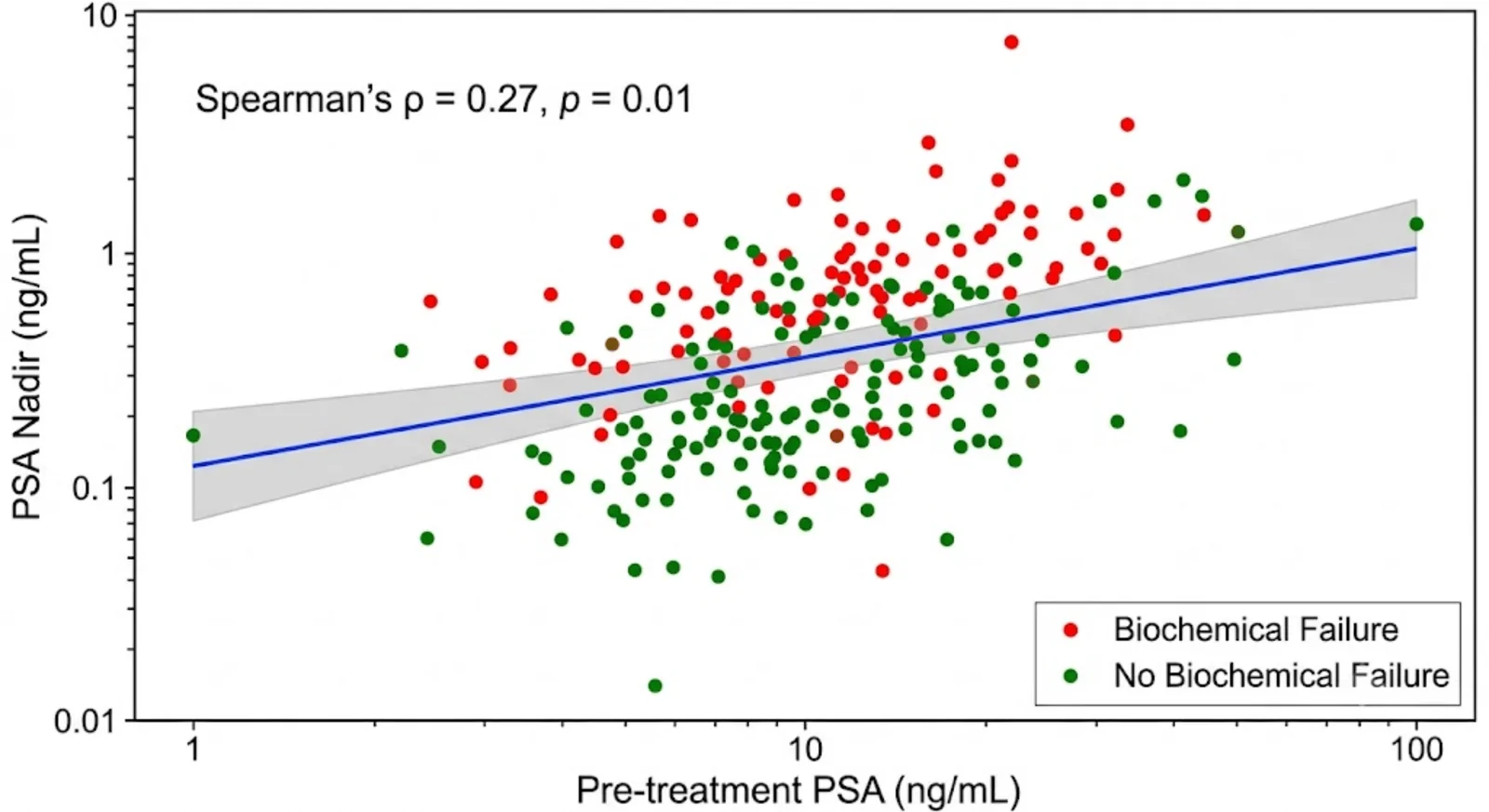
Scatter Plot of Pre-treatment PSA vs. PSA Nadir This scatter plot illustrates the correlation between pre-treatment PSA (x-axis, log scale) and PSA nadir (y-axis, log scale). Each point stands for a different patient. The regression line shows a weak but statistically significant positive correlation (Spearman's ρ = 0.27, p = 0.01), which means that patients whose PSA levels were higher at the start tend to have higher nadir values. Points are color-coded by biochemical failure status (red = biochemical failure, green = no biochemical failure). PSA: prostate-specific antigen

## Discussion

This study provides the first comprehensive evaluation of biochemical failure patterns following definitive radiotherapy for localized prostate cancer in a Jordanian military population. The biochemical failure rate of 22 out of 91 (24.2%) over a median follow-up of 36 months, along with two- and five-year bRFS rates of 84.6% and 70.3%, respectively, indicates that modern radiotherapy meets international standards [19,20].

Pre-treatment PSA as a predictor

Pre-treatment PSA greater than 20 ng/mL emerged as a strong independent predictor of biochemical failure, with an HR of 2.8. This finding is consistent with the seminal risk stratification system proposed by D'Amico et al., which identified PSA greater than 20 ng/mL as a defining feature of high-risk disease [7]. Contemporary series incorporating dose-escalated radiotherapy and ADT have reported five-year bRFS rates of 60%-70% for high-risk patients [21], closely matching our observed rate of 64.5% in this subgroup.

PSA nadir as a post-treatment biomarker

The identification of PSA nadir greater than 0.1 ng/mL as the strongest predictor of biochemical failure (HR: 3.2) represents a clinically actionable finding. This threshold has been validated across multiple studies [12,22]. Patients achieving a nadir of ≤0.1 ng/mL had a favorable prognosis, with a 3.2-fold lower hazard of subsequent biochemical failure compared to those with a nadir of >0.1 ng/mL, supporting its use as a treatment response biomarker. The higher hazard associated with PSA nadir > 0.1 ng/mL may indicate residual microscopic disease, more aggressive tumor biology, or an incomplete response to therapy.

Stage-wise radiotherapy and ADT considerations

Our cohort's risk-adapted approach to radiotherapy dosing and ADT duration follows current guidelines, with higher doses and longer ADT for patients at high or very high risk (Table 1). All patients received conventional fractionation using IMRT/VMAT; SBRT and hypofractionated regimens were not used in this cohort. The observation that advanced-stage patients (high/very high risk) had higher biochemical failure rates (64.5% five-year bRFS) compared to intermediate-risk (84.0%) and low-risk (100%) patients is consistent with the known biology of prostate cancer, where more advanced disease carries a higher risk of recurrence despite intensified treatment.

Clinical implications

These findings have several practical applications. Risk-adapted follow-up protocols can be developed based on pre-treatment PSA and post-treatment nadir response. Patients at high or very high risk need more careful monitoring during the first 36 months, when most biochemical failure events happen. Patients with a nadir above 0.1 ng/mL should be identified for closer monitoring and earlier consideration of salvage strategies. Institution-specific benchmarks enable evidence-based patient counseling regarding expected outcomes. However, these findings require prospective validation in larger, multicenter cohorts before individualized surveillance strategies can be implemented more widely [23,24].

Limitations

This study has several limitations. First, the retrospective, single-center design may limit generalizability and introduce the potential for selection bias. Second, the modest sample size (n = 91) and limited number of biochemical failure events (n = 22) restricted statistical power for some subgroup analyses. Third, the median follow-up of 36 months precludes analysis of long-term endpoints, including distant metastasis and prostate cancer-specific mortality, and the five-year bRFS estimates should be interpreted with caution given the limited number of patients with extended follow-up. Fourth, variation in radiation techniques and fractionation schedules may introduce confounding. Fifth, the very high use of ADT (97.8%) may influence PSA nadir values and biochemical failure rates, potentially limiting the generalizability of our findings to settings where ADT is used less frequently. Sixth, the dataset lacked detailed information on patient comorbidities and quality of life outcomes. Seventh, while temporal validation supports internal validity, external validation in other Jordanian hospitals and international cohorts is necessary before widespread adoption.

Future research directions

Future research should focus on prospective validation of these findings in larger, multicenter cohorts to confirm the generalizability of our results across diverse populations and clinical settings. Longer follow-up is needed to assess the relationship between biochemical failure and clinical endpoints such as distant metastasis and overall survival and to validate the stability of five-year survival estimates. Incorporating novel biomarkers, including genomic classifiers and advanced imaging, may further refine risk stratification. Multicenter studies with standardized follow-up protocols would enable the development of evidence-based surveillance schedules tailored to individual patient risk profiles, optimizing resource utilization and ensuring timely detection of disease progression.

## Conclusions

Definitive radiotherapy achieves favorable biochemical control rates in this military cohort, comparable to international benchmarks. Pre-treatment PSA greater than 20 ng/mL and post-treatment PSA nadir greater than 0.1 ng/mL emerged as the strongest independent predictors of biochemical failure. These readily available parameters support dynamic risk stratification and personalized follow-up protocols. Patients achieving a nadir of 0.1 ng/mL or lower have a favorable prognosis with a lower hazard of subsequent biochemical failure, while those with incomplete biochemical response warrant intensive surveillance and early consideration of salvage strategies. However, these findings require prospective validation in larger, multicenter cohorts before individualized surveillance strategies can be widely implemented.
